# Fenofibrate induces apoptosis of triple-negative breast cancer cells via activation of NF-κB pathway

**DOI:** 10.1186/1471-2407-14-96

**Published:** 2014-02-16

**Authors:** Ting Li, Qunling Zhang, Jian Zhang, Gong Yang, Zhimin Shao, Jianmin Luo, Minhao Fan, Chen Ni, Zhenhua Wu, Xichun Hu

**Affiliations:** 1 Department of Medical Oncology, Fudan University Shanghai Cancer Center, 200032 Shanghai, China; 2 Department of Oncology, Shanghai Medical College, Fudan University, 200032 Shanghai, China; 3 Department of Breast Surgery, Fudan University Shanghai Cancer Center, 200032 Shanghai, China; 4 Department of Cancer Research Laboratory, Fudan University Shanghai Cancer Center, 200032 Shanghai, China

**Keywords:** Triple-negative breast cancer, Fenofibrate, Anti-proliferation, Apoptosis, NF-κB, Cell cycle arrest

## Abstract

**Background:**

There are a lot of unmet needs in patients with triple-negative breast cancer (TNBC). Fenofibrate, a peroxisome proliferator-activated receptor alpha (PPAR-α) agonist, has been used for decades to treat hypertriglyceridaemia and mixed dyslipidaemia. Recent studies show that it might have anti-tumor effects, however, the mechanism remains unclear. Here, we assessed the ability of fenofibrate to induce apoptosis of TNBC *in vitro* and *in vivo* and explored involved mechanisms.

**Methods:**

MTT method was used to evaluate the anti-proliferation effect of fenofibrate, and invert microscope to observe the apoptotic morphological changes. The percentage of apoptotic cells and distribution ratios of cell cycle were determined by flow cytometric analysis. The related protein levels were measured by Western blot method. The changes of genes and pathways were detected by gene expression profiling. The tumor growth *in vivo* was assessed by MDA-MB-231 xenograft mouse model. Terminal deoxytransferase-catalyzed DNA nick-end labeling (TUNEL) assay was employed to estimate the percentage of apoptotic cells *in vivo*. In order to evaluate the safety of fenofibrate, blood sampled from rat eyes was detected.

**Results:**

We found that fenofibrate had anti-proliferation effects on breast cancer cell lines, of which the first five most sensitive ones were all TNBC cell lines. Its induction of apoptosis was independent on PPAR-α status with the highest apoptosis percentage of 41.8 ± 8.8%, and it occurred in a time- and dose-dependent manner accompanied by up-regulation of Bad, down-regulation of Bcl-xl, Survivin and activation of caspase-3. Interestingly, activation of NF-κB pathway played an important role in the induction of apoptosis by fenofibtate and the effect could be almost totally blocked by a NF-κB specific inhibitor, pyrrolidine dithiocarbamate (PDTC). In addition, fenofibrate led to cell cycle arrest at G0/G1 phase accompanied by down-regulation of Cyclin D1, Cdk4 and up-regulation of p21, p27/Kip1. *In vivo*, fenofibrate slowed down tumor growth and induced apoptosis with a good safety profile in the MDA-MB-231 xengograft mouse model.

**Conclusions:**

It is concluded that fenofibrate induces apoptosis of TNBC via activation of NF-κB pathway in a PPAR-α independent way, and may serve as a novel therapeutic drug for TNBC therapy.

## Background

Breast cancer is the most common malignant cancer in women globally. Based on different gene expression profiles, breast cancer is classified into at least four subtypes [1]. Triple-negative breast cancer (TNBC) is a special subtype of breast cancer, which is defined as the absence of estrogen and progesterone receptor expression as well as ERBB2 amplification. Therefore, when compared with other subtypes of breast cancer, TNBC has no response to endocrine or anti-ERBB2 therapies and systemic chemotherapy is the major treatment for those patients after metastasis. However, there is no standard therapeutic regimen up to now, and a vast majority of deaths occur in the first 5 years after treatment [2], making that TNBC as a whole group still has a poor outcome. Therefore, new effective and safe drugs are urgently needed to be found.

Fenofibrate is a fibric acid derivative and plays an important role in lowering the levels of serum cholesterol and triglyceride and elevating the levels of high density lipoproteins [3]. It has been used for years to treat severe hypertriglyceridaemia and mixed dyslipidaemia through activating of peroxisome proliferator-activated receptor-α (PPAR-α) [3], which is a specific transcription factor belonging to the nuclear receptor superfamily [4].

Recent studies showed that fenofibrate might have anti-tumor effects, however, the detailed mechanisms were not fully understood. Although such anti-tumor effects were present in B-cell lymphoma [5], prostate cancer [6], glioblastoma [7], mantle cell lymphoma [8], squamous cell carcinoma [9], hepatocellular carcinoma [10,11], glioma [12], melanoma [13,14], lung cancer [13,15], fibrosarcoma [13], medulloblastoma [16] and endometrial cancer [17], the effects of fenofibrate on breast cancer, especially on TNBC had not been reported yet. Murad et al. [18] just showed that treatment with fenofibrate decreased the semaphorin 6B gene expression of breast cancer cells, which had a broad range of functions, from immune response and cell migration to angiogenesis and cancer.

A better understanding of the effects and mechanisms may shed light on the new potential TNBC therapy. Therefore, we assessed the anti-tumor effects of fenofibrate in breast cancer cell lines and then explored the possible mechanisms involved.

## Methods

### Reagents and antibodies

3-(4, 5-dimethylthiazol-2-yl) -2, 5-diphenyltetrazolium bromide (MTT), pyrrolidine dithiocarbamate (PDTC), fenofibrate and giemsa stain were purchased from Sigma (St Louis, MO, USA). GW6471 was purchased from Tocris Bioscience (Ellisville, MO, USA). Beta-Actin, p21, p27/Kip1, Cyclin D1, Akt1, Phospho Akt1 (pS473), Phospho Erk1 (pT202) / Erk2 (pT185), NF-κB (p65subunit), IKK-α, IκBα and Phospho-IκBα (pS36) antibodies were purchased from Epitomics (Burlingame, CA, USA). Cdk2, Cdk4, Cdk6, p53 (DO-2) and TFIIB (D-3) antibodies were purchased from Santa Cruz Biotechnology (Santa Cruz, CA, USA). CyclinB1, Bad (D24A9), Bid, Bcl-2, Bcl-xl, Survivin (71G4B7), Caspase3, Erk1/2 and Phospho-IKKα(Ser176)/IKK β(Ser177) antibodies were purchased from Cell Signaling Technology (Beverly, MA, USA). PE Annexin V Apoptosis Detection Kit I was purchased from BD Bioscience (San Jose, CA,USA). Cell cycle staining solution was purchased from MultiSciences Biotech (Hangzhou, China). Terminal deoxytransferase-catalyzed DNA nick-end labeling (TUNEL) assay was from Promega Corporation (Madison, WI, USA). The gene expression profile was done by KangChen Biotechnology Company (Shanghai, China).

### Cell culture

Cell lines including SK-BR-3, MCF-7, T47D, HCC1937, HS578T, MDA-MB-231, MDA-MB-436, BT549, MDA-MB-453, MDA-MB-468 and MCF-10A cells were purchased from the American Type Culture Collection (ATCC, Bethesda, MD, USA). All cell lines had been tested and authenticated by ATCC. In brief, morphology and proliferation of cells were routinely assessed and the identities of cells were verified by isoenzyme and short tandem repeat analysis. Cells were also regularly tested for mycoplasma infection. MDA-MB-231HM and MDA-MB-231-B cell lines were established by our institute according to previously described method [19]. The MDA-MB-231HM cell line had a high potential to metastasize to lung and MDA-MB-231-B cell line was obtained from bone metastases resulting from MDA-MB-231. All cell lines were used for no more than 3 months after being thawed.

Breast cancer cell lines were cultured in the ATCC-recommended media, which were supplemented with 10% fetal bovine serum. Cells were cultured as a monolayer in 5% CO2 and 95% air in a humidified incubator at 37°C and collected during their exponential growth phase. Cells were cultured for 24 hours till attachment before experimental use.

### Cell proliferation analysis

Cells were seeded into 96-well tissue culture plates (Nunclon™) at a density of 3 × 10^4^ cells/mL in a volume of 180 μL culture media and treated with various conditions for different duration of time. Each well was added with 20 μL of MTT reagent (0.5 mg/mL) and incubated at 37°C for 4 hours. Afterwards, the supernatant was sucked out, and the same volume of dimethyl sulfoxide (DMSO) was added to each well to dissolve the resulting formazan crystals at 37°C for 20 min. The optical density values (OD value) were measured at 490 nm using a plate reader (BioTek Company). The inhibition ratios for each treatment condition were calculated by OD values. The potency of cell proliferation inhibition was expressed as a half maximal inhibitory concentration (IC_50_) value.

### Cell staining analysis

MDA-MB-231 cells were seeded into 6-well tissue culture plates (Corning) at a density of 1 × 10^5^ cells/mL in a volume of 2 mL culture media and treated with fenofibrate for 24 hours. The plates were washed with PBS once and cells were fixed with cold methanol for 10 minutes. After washed twice with PBS, cells were stained with Giemsa staining solution, observed and photographed under the microscope.

### Apoptosis analysis

Apoptosis was detected by PE Annexin V Apoptosis Detection Kit I according to the manual instruction. In brief, cells were washed with PBS twice and 1 × Binding Buffer once and then suspended in 1 × Binding Buffer. Cells were double-stained with PE Annexin V and 7-AAD for 15 minutes in the dark at room temperature, and then analyzed by flow cytometry.

### Cell cycle analysis

Cells were harvested and washed with cold PBS, and then fixed with 75% ethanol at -20°C overnight. The fixed cells were washed with cold PBS twice, added 500 μL DNA staining solution (including 200 μg/mL RNase A and 20 μg/mL propidium iodide staining solution) and incubated for 30 minutes. Finally, cells were analyzed by flow cytometry in the presence of the dye.

### Western blot analysis

Western blot analysis was performed according to the method described previously [20]. Briefly, cell lysates were added and proteins from each group were extracted, separated by standard SDS-PAGE and then transferred onto polyvinylidene difluoride membranes. The membranes were washed, blocked and incubated with specific primary antihuman antibodies at 4°C overnight. Afterwards, the membranes were washed and incubated by horseradish peroxidase-conjugated secondary antibodies for 1 hours at room temperature. The signals were visualized by luminescent image analyzer (ImageQuant LAS4000 mini). TFIIB [21] and β-actin were detected as a loading control.

### Human expression microarray analysis

The total RNA was extracted by TRIzol after harvesting cells treated with fenofibrate. The Whole Human Genome Oligo Microarray (4x44K, Agilent Technologies) was done by KangChen Biotechnology. The data extracted from Agilent Feature Extraction software (version 11.0.1.1) were quantile normalized and analyzed by the GeneSpring GX v11.5.1 software package (Agilent Technologies). The fold change filtering identified differentially expressed genes. Pathway and gene ontology (GO) analysis were applied to identify the roles of these differentially expressed genes playing in biological pathways or GO terms. The microarray data was accessible through Gene Expression Omnibus (GEO) [22] series accession number GSE49965 (http://www.ncbi.nlm.nih.gov/geo/query/acc.cgi?acc=GSE49965).

### Nude mouse xenograft model of human tumor

Six-week-old female BALB/c nude mice (Laboratory Animal Center of Chinese Academy of Sciences, Shanghai Branch) were used. Xenografts were initiated by subcutaneous injection of 2 × 10^6^ MDA-MB-231 cells into each mouse (n = 10 for each group). Seven days after injection, 200 mg/kg of fenofibrate suspended in 5% sodium carboxymethylcellulose were given daily via intragastric administration in treatment group, while the equal volume of 5% sodium carboxymethylcellulose was administrated in the control group. The treatment lasted 21 days. The tumor volume was measured every three days and calculated in the following formula: length × width × height/2 [23]. At the end of the study, tumors were carefully removed and the paraffin sections were prepared for TUNEL analysis. Blood was sampled from the eyes of all mice and detected. All procedures for animal care were approved by the Animal Management Committee of Fudan University.

### TUNEL assay

The DeadEnd™ Colorimetric TUNEL System was from Promega Corporation (USA) and used according to manufacturer’s instructions.

### Statistical analysis

Variance between the groups was analyzed using a two-tailed t-test. P < 0.05 was considered to be significant. All statistical analyses were performed using SPSS 16.0 software.

## Results

### Inhibition of cell proliferation

**Figure 1:**
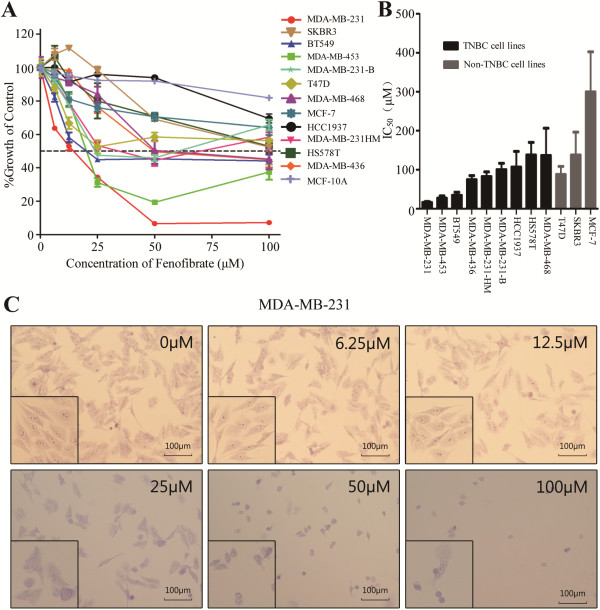
**Fenofibrate inhibits breast cancer cells proliferation. (A)** Fenofibrate was able to inhibit the proliferation of twelve breast cancer cell lines in a dose-dependent manner, but had the least effect on MCF-10A cells. **(B)** The 72 h IC_50_ for each cell line was shown and the first five most sensitive cell lines to fenofibrate were all TNBC cell lines, that were MDA-MB-231, MDA-MB-453, BT549, MDA-MB-436 and MDA-MB-231HM cells, and MDA-MB-231 cells were the most sensitive ones. Data represent the means ± SD of three independent experiments. TNBC = triple-negative breast cancer. **(C)** A considerable number of MDA-MB-231 cells had morphological changes, including the shrinkage and rounding up of cells when treated with fenofibate.

In order to verify the anti-cancer effects of fenofibrate on the cell lines representing different molecular subtypes, twelve breast cancer cell lines and one human breast epithelial cells, MCF-10A, were treated with fenofibrate at different concentrations (0, 6.25, 12.5, 25, 50 and 100 μΜ, DMSO in each group was balanced) for 72 hours. Figure 1A showed that fenofibrate inhibited the proliferation of the twelve breast cancer cell lines in a dose-dependent manner, especially of TNBC cell lines, but had the least effect on MCF-10A cells. The first five most sensitive ones were all TNBC cell lines, that were MDA-MB-231, MDA-MB-453, BT549, MDA-MB-436 and MDA-MB-231HM cell lines, and their IC_50_ for 72 hours were 16.07 ± 4.44 μM, 26.72 ± 10.04 μM, 34.47 ± 13.88 μM, 74.46 ± 17.75 μM and 82.09 ± 21.21 μM respectively, and MDA-MB-231 cells were the most sensitive ones (Figure 1B). Fenofibrate inhibited the proliferation of T47D, MCF-7 and SKBR3 cells, however, when compared with TNBC cell lines, they were comparatively less responsive and their IC_50_ were all above 80 μM (Figure 1B). Therefore, we chose MDA-MB-231 cells as a representative for the subsequent study.

Figure 1C showed that as early as 24 hours after fenofibrate treatment at different concentrations (0, 6.25, 12.5, 25, 50, and 100 μM, DMSO in each group was balanced), the number of MDA-MB-231 cells decreased and morphology was altered with features, that were the shrinkage and rounding up of cells.

### Induction of apoptosis

**Figure 2:**
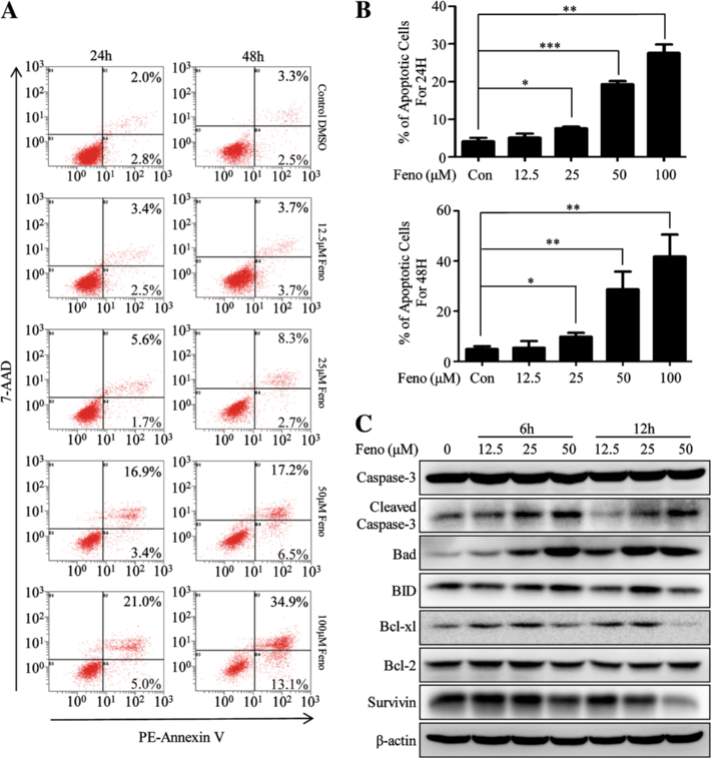
**Fenofibrate induces apoptosis of MDA-MB-231 cells. (A, B)** Fenofibrate induced apoptosis of MDA-MB-231 cells in a time- and dose-dependent manner. **(C)** Western blot analysis confirmed that fenofibrate dramatically up-regulated the expression of Bad and down-regulated the expressions of Bcl-xl and Survivin and finally induced cleavage of caspase-3, but had no impact on BID and Bcl-2. **(A, C) **All the experiments were repeated three times and the representative ones of those results were shown. **(B)** Data represent the means ± SD of three independent experiments. Feno = fenofibrate, Con = control, *indicates p < 0.05, **indicates p < 0.01, ***indicates p < 0.001.

In order to elucidate the detailed mechanisms of death induced by fenofibrate in MDA-MB-231 cells, we did further experiments. MDA-MB-231 cells were treated with fenofibrate at different concentrations (0, 12.5, 25, 50, and 100 μM, DMSO in each group was balanced) for 24 and 48 hours. As shown in Figure 2A and B, the percentage of apoptotic cells reached 27.6 ± 2.2% and 41.8 ± 8.8% after 24 and 48 hours incubation with 100 μM fenofibrate, increasing by an almost 6.7- and 8.4-fold respectively when compared with DMSO-treated cells, suggesting a dose- and time-dependent manner. Besides MDA-MB-231 cells, fenofibrate induced apoptosis of BT549 cells and had little effect on MCF-10A cells (see Additional file 1A and B).

Next we explored how fenofibrate mediated the apoptosis-inducing effect on MDA-MB-231 cells. Given that Bad, BID, related to the apoptosis-promoting process, and Bcl-xl, Bcl-2, Survivin, related to the apoptosis-inhibiting process, were key regulators of apoptosis, we investigated the effects of fenofibrate on these protein expressions. The whole cell extracts from MDA-MB-231 cells exposed to fenofibrate in various concentrations (0, 12.5, 25 and 50 μM, DMSO in each group was balanced) for 6 hours and 12 hours were detected by Western blot. On one hand, Bad was dramatically up-regulated, which might explain the prominent apoptosis-inducing capacity of fenofibrate. No significant change of BID was found for both 6 hours and 12 hours treatments. On the other hand, Bcl-xl and Survivin were significantly down-regulated, and fenofibrate had no effect on the Bcl-2 level. Furthermore, we found activation of caspase-3 (Figure 2C). All the results provided supports for our findings. In short, fenofibrate induced apoptosis of MDA-MB-231 cells through enhancing the expression of Bad and decreasing the expressions of Bcl-xl and Survivin, and finally resulting in activation of caspase-3.

### Cell cycle alteration

**Figure 3:**
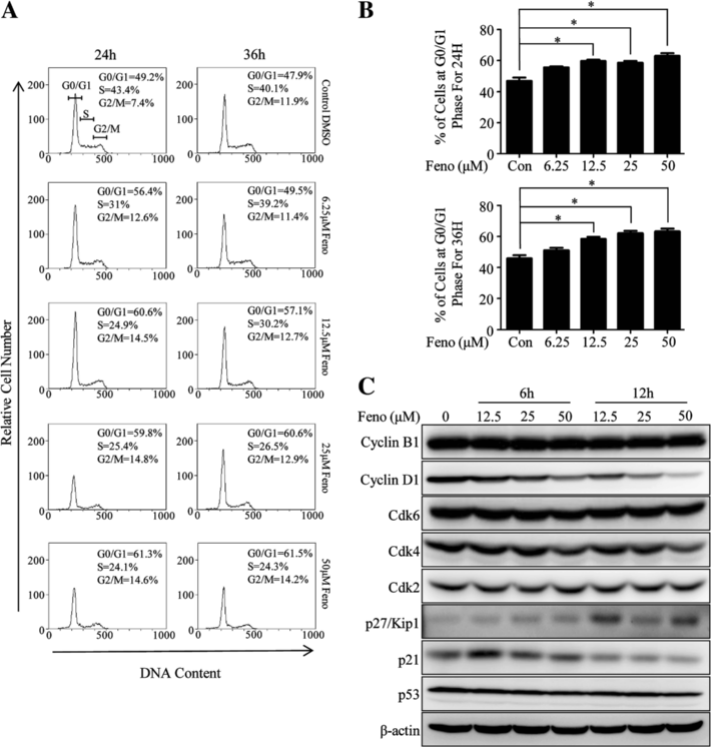
**Fenofibrate alters cell cycle of MDA-MB-231 cells. (A, B)** Treatment with fenofibrate led to cell cycle arrest of MDA-MB-231 cells at G0/G1 phase. **(C)** Fenofibrate exposure caused a time- and dose-dependent decrease of Cyclin D1 and Cdk4 and increase of p21 and p27/Kip1 while the expressions of p53, Cdk2, Cdk6 and Cyclin B1 remained unchanged. **(A, C)** All the experiments were repeated three times and the representative ones of those results were shown. **(B)** Data represent the means ± SD of three independent experiments. Feno = fenofibrate, Con = control, *indicates p < 0.05.

To further examine that whether cell cycle arrest was responsible for proliferation inhibition induced by fenofibrate, MDA-MB-231 cells were treated with various concentrations (0, 6.25, 12.5, 25 and 50 μM, DMSO in each group was balanced) of fenofibrate for 24 and 36 hours and examined by flow cytometry. The percentages of cells at G0/G1 phase were only 47.0 ± 3.0% for 24 hours and 45.9 ± 2.9% for 36 hours in the control group, and they increased to 63.0 ± 2.4% and 63.3 ± 2.6% respectively when the concentration of fenofibrate reached 50 μM and the effect was weaker when other concentrations were given (Figure 3A and B). The similar cell cycle arrest was found in MDA-MB-468 cells (see Additional file 1C).

To determine how fenofibrate led to cell cycle arrest at G0/G1 phase, the whole cell extracts from MDA-MB-231 cells exposed to fenofibrate of various concentrations (0, 12.5, 25 and 50 μM, DMSO in each group was balanced) for 6 and 12 hours were detected by Western blot. In line with our cell cycle results analyzed by flow cytometry, the expression levels of Cyclin D1 and Cdk4, which were the G0/G1 phase related proteins promoting cell cycle progress, were decreased in a time- and dose-dependent manner (Figure 3C), when compared with DMSO- treated cells. As expected, the levels of p21 and p27/Kip1, whose effects were opposite to that of cyclin D1 and Cdk4, were increased (Figure 3C). There were no significant changes of p53, Cdk2, Cdk6 and Cyclin B1. All data demonstrated that treatment with fenofibrate led to cell cycle arrest of MDA-MB-231 cells at G0/G1 phase.

### Cell proliferation inhibition and apoptosis inducement independent of PPAR-α

**Figure 4:**
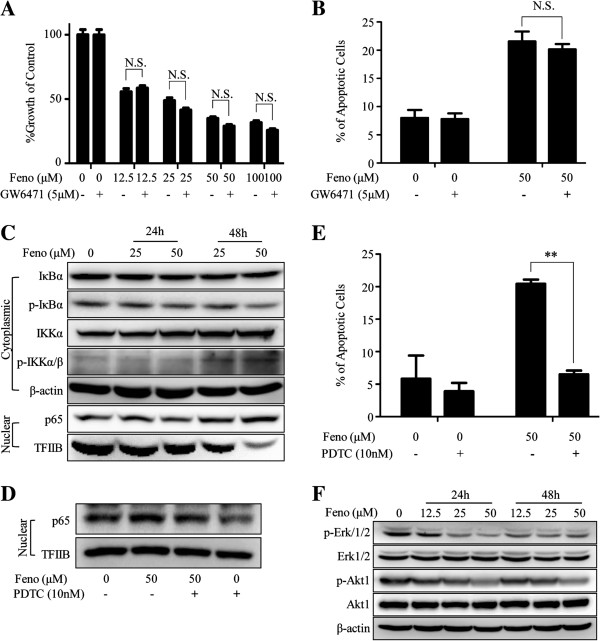
**Fenofibrate induces apoptosis via activation of NF-κB pathway in a PPAR-α independent way.** GW6471 (5 μM), a PPAR-α specific inhibitor, did not provide protection from anti-proliferation **(A)** and apoptosis **(B)** induced by fenofibrate. Data represent the means ± SD of three independent experiments. **(C)** Fenofibrate induced nuclear accumulation of p65 accompanied by increasing the levels of p-IKKα/β and IKKα and decreasing the level of p-IκBα, but had no impact on IκBα. **(D)** PDTC (10nM), a specific inhibitor of NF-κB, efficiently inhibited the nuclear accumulation of p65 induced by fenofibrate. **(E)** Cell apoptosis induced by fenofibrate was significantly decreased by co-treatment with fenofibrate and 10nM PDTC (** indicates p < 0.01 relative to fenofibrate treatment group). Data represent the means ± SD of three independent experiments. **(F)** Exposure to fenofibrate triggered a decrease in p-Akt1 and p-Erk1/2, but had no effects on Akt1 and Erk1/2. TFIIB was detected as a loading control of nuclear proteins. β-actin was served as a loading control of cytoplasmic proteins and whole cell proteins. N.S. = No statistical significance. Feno = fenofibrate.

Fenofibrate exerts the effect of lowering the levels of serum lipids over the activation of PPAR-α. MDA-MB-231 cells also express PPAR-α [24], so the question whether PPAR-α mediates anti-tumor effects of fenofibrate on MDA-MB-231 cells should be answered. GW6471 is a PPAR-α specific inhibitor with a median inhibitory concentration of 0.24 μM [25], and it is reported that 1.6 μM GW6471 inhibited the transcriptional activity of endogenous PPAR-α [10]. Furthermore, Additional file 2 showed that 5 μM GW6471 effectively inhibited the PPAR-α classic target gene expression of MDA-MB-231 cells (see Additional file 2 and Additional file 3). Therefore, 5 μM GW6471 was added to inhibit PPAR-α. As shown in the Figure 4A, the growth ratio of fenofibrate alone (0, 12.5, 25, 50 and 100 μM, DMSO in each group was balanced) vs. fenofibrate in combination with 5 μM GW6471 in 72 hours were 100.00 ± 9.14% vs. 99.90 ± 9.23%, 55.74 ± 5.43% vs. 58.60 ± 4.10%, 48.76 ± 5.16% vs. 41.43 ± 3.66%, 34.97 ± 2.82% vs. 28.92 ± 2.94%, 31.69 ± 3.43% vs. 25.71 ± 2.84% respectively, p > 0.05. In addition, the percentage of apoptotic cells treated with 50 μM fenofibrate alone vs. 50 μM fenofibrate in combination with 5 μM GW6471 in 24 hours was 21.55 ± 2.47% vs. 20.15 ± 1.34%, p > 0.05 (Figure 4B). The results above indicated that the drug might mediate the anti-cancer effects in a way independent of PPAR-α status.

### Fenofibrate induces apoptosis through activation of NF-κB pathway

Since apoptosis induced by fenofibrate was independent of PPAR-α, further investigation about the apoptosis mechanism was performed. Given that NF-κB was well known for its significant role in apoptosis, we detected the levels of its pathway related proteins and their phosphorylation status. NF-κB (p65) is inactive in the cytoplasm where it combines with IκB, mainly IκBα, which is regulated by IKKα/β. Under some stimuli, IκBα is phosphorylated by IKKα/β, then undergoes ubiquitination and degradation to release p65. Afterwards, p65 translocates to nucleus and promotes the transcriptions of target genes. As shown in Figure 4C, in MDA-MB-231 cells, the nuclear p65, the most abundant form of NF-κB, increased after fenofibrate treatment (0, 25 and 50 μM, DMSO in each group was balanced) for 24 and 48 hours, accompanied by up-regulation of phosphor-IKKα/β and IKKα and down-regulation of phosphor-IκBα in cytoplasm, but IκBα remained unchanged. All data showed that activation of NF-κB pathway was present with fenofibrate treatment.

In the next step, we explored that whether activation of NF-κB pathway contributed to the apoptosis effect induced by fenofibrate. PDTC is a specific inhibitor of NF-κB, which blocks the transactivation of NF-κB by suppressing the release of inhibitory subunit IκB from the cytoplasmic form of NF-κB [26]. As shown in Figure 4D, in comparison with 50 μM fenofibrate treatment, the nuclear p65 decreased under 10nM PDTC treatment alone or in combination with 50 μM fenofibrate for 48 hours in MDA-MB-231 cells. As shown in Figure 4E, the percentages of apoptotic cells were 5.85 ± 5.02% for control and 3.90 ± 1.84% for the 10 nM PDTC treatment alone (p > 0.05). However, compared with 50 μM fenofibrate treatment alone, it significantly decreased from 20.45 ± 0.92% to 6.50 ± 0.85% when treated with 50 μM fenofibrate in combination with 10nM PDTC for 24 h in MDA-MB-231 cells (p < 0.01). These results confirmed that activation of NF-κB pathway accounted for the apoptosis effect induced by fenofibrate.

In addition, we also explored the functions of Akt1 and Erk1/2 pathways in anti-tumor effects of fenofibrate. Figure 4F showed a down-regulation of phosphorylation of Akt1 and Erk1/2, but no changes occurred in the total expressions of Akt1 and Erk1/2 after fenofibrate treatment (0, 12.5, 25 and 50 μM, DMSO in each group was balanced) for 24 and 48 hours in MDA-MB-231 cells. Therefore, Akt1 and/or Erk1/2 signaling pathways might also be involved in the anti-tumor effects of fenofibrate in MDA-MB-231 cells.

### The gene expression profile

**Figure 5:**
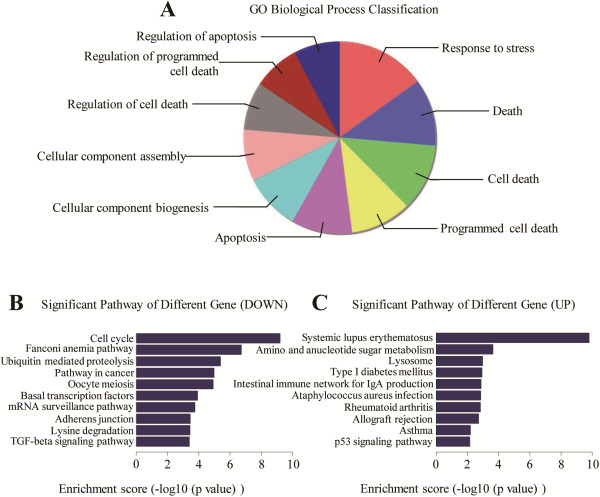
**Changes of the gene expression profiling.** After fenofibrate treatment, **(A)** the top ten most obvious changes in GO biological process classification confirmed that fenofibrate could induce apoptosis of MDA-MB-231 cells. **(B)** Cell cycle pathway ranked first in the top ten most significant down-regulated pathways. **(C)** p53 pathway ranked tenth in the top ten most significant up-regulated pathways.

To make further investigation of the apoptosis-inducing effects of fenofibrate, we used the gene expression profile chip to compare the changes between the control group (0 μM for 24 hours) and fenofibrate treatment group (50 μM for 24 hours) in MDA-MB-231 cells. As shown in Figure 5A, the top ten most obvious changes in GO biological process classification were response to stress, death, cell death, programmed cell death, apoptosis, cellular component biogenesis, cellular component assembly, regulation of cell death, regulation of programmed cell death and regulation of apoptosis, out of which 7 were related to death, 4 to apoptosis. In the top ten most significant down-regulated pathways, cell cycle ranked first and pathway in cancer ranked fourth (Figure 5B). In the top ten most significant up-regulated pathways, p53 pathway ranked tenth (Figure 5C). These data was in line with our results *in vitro*.

### Slowing down tumor growth and induction of apoptosis *in vivo*

**Figure 6:**
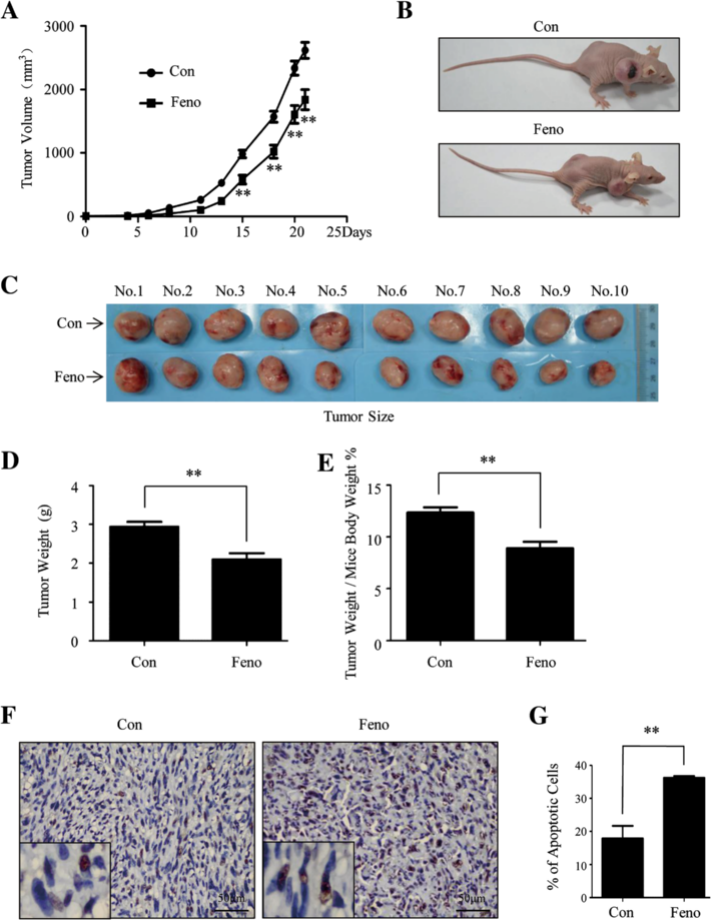
**Fenofibrate slow down the growth of xenograft model of triple-negative breast cancer by inducing apoptosis. (A)** Tumor volume with days after treatment. The volumes of tumors in the two groups reached a significant difference 15 days after fenofibrate treatment (** indicates p < 0.01 relative to the control group). **(B, C)** The tumor sizes in the treatment group were significantly smaller than that of the control group at the end of the study. **(D, E)** The tumor weight and the percentage of tumor weight/ mice body weight of the treatment group were significantly lighter than that of the control group (** indicates p < 0.01 relative to the control group). **(F, G)** The analysis of paraffin-embedded human breast cancer tissue from nude mouse models by TUNEL assay. Compared with the control group, TUNEL assay showed an increased number of apoptotic cells in fenofibrate treatment group (** indicates p < 0.01 relative to the control group). Feno = fenofibrate, Con = control.

We further explored the effect of fenofibrate on tumor growth *in vivo*. As shown in Figure 6A, the volumes of tumors in the two groups reached the significant difference after 15 days of fenofibrate treatment (984.11 ± 59.99 mm^3^ for control and 578.79 ± 70.44 mm^3^ for fenofibrate on day 15, p < 0.01). The tumor sizes, weight of tumors and the percentage of tumor weight/mice body weight in the treatment group were significantly smaller than those of the control group after 21 days of fenofibrate treatment (2.09 ± 0.16 g vs. 2.94 ± 0.13 g for tumor weight and 8.89 ± 0.64% vs. 12.34 ± 0.52% for the percentage of tumor weight/mice body weight, p < 0.01, Figure 6B, C, D and E).

In order to confirm that the effect on tumor growth *in vivo* was due to apoptosis induced by fenofibrate, the TUNEL assay was carried out. Compared with the control group, Figures 6F and G showed that the percentage of apoptotic cells with treatment increased from 17.84 ± 6.63% to 36.22 ± 0.87% (p < 0.01).

**Figure 7:**
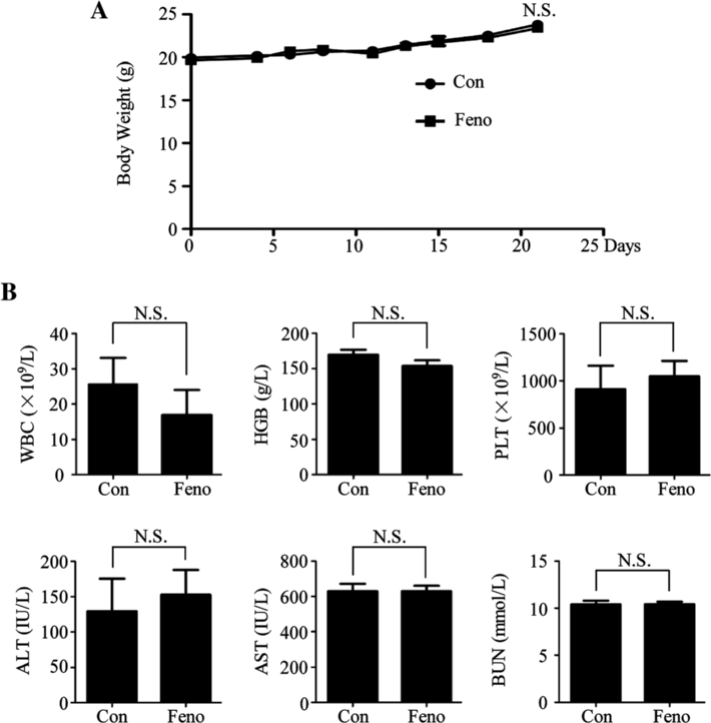
**Fenofibrate has a good safety profile.** No obvious treatment-related toxicity in terms of body weight **(A)**, hematopoietic function, liver function and kidney function of animals **(B)** was observed. Con = control group, Feno = fenofibrate group, WBC = white blood cells, HGB = hemoglobin, PLT = platelet, AST = aspartate aminotransferase, ALT = alanine transaminase, BUN = blood urea nitrogen, N.S. = No statistical significance.

The safety of fenofibrate was also evaluated *in vivo*. As shown in the Figure 7A and B, there were no statistical differences between the control and treatment groups in body weight (23.80 ± 1.25 g vs. 23.40 ± 1.30 g, p > 0.05), white blood cells (WBC, 25.76 ± 7.36 × 10^9^/L vs. 16.93 ± 7.08 × 10^9^/L, p > 0.05), hemoglobin (HGB, 169.70 ± 7.04 g/L vs. 153.78 ± 7.92 g/L, p > 0.05), platelet (PLT, 911.00 ± 249.70 × 10^9^/L vs. 1048.67 ± 163.30 × 10^9^/L, p > 0.05), alanine transaminase (ALT, 129.44 ± 46.12 IU/L vs. 152.77 ± 35.09 IU/L, p > 0.05), aspartate aminotransferase (AST, 629.57 ± 42.23 IU/L vs. 630.21 ± 29.93 IU/L, p > 0.05) and blood urea nitrogen (BUN,10.41 ± 0.39 mmol/L vs. 10.44 ± 0.25 mmol/L, p > 0.05), suggesting that fenofibrate was safe and had little side effects on hematologic, hepatic and renal function *in vivo*. These results showed that fenofibrate slowed down tumor growth and induced apoptosis in xenograft mouse model with a good safety profile.

## Discussion

To our best knowledge, the present study first showed the activity of fenofibrate against TNBC cell lines both *in vitro* and *in vivo*. Our results showed that the involved mechanisms resulted from the convergent effects on cell apoptosis mediated by NF-κB nuclear translocation and subsequent transactivation and cell cycle arrest by fenofibrate treatment.

Caspase plays a central role in the execution of apoptosis, especially caspase-3, and a variety of apoptotic signaling would lead to activation of caspase-3 [27]. The increase of pro-apoptosis Bcl-2 family, such as Bad and BID, decrease of anti-apoptosis Bcl-2 family, such as Bcl-xl and Bcl-2, and down-regulation of Survivin [28] led to apoptosis by activation of caspase-3. Study showed that fenofibrate induced apoptosis in mantle cell lymphoma followed by caspase-3 activation [8]. The Bcl-2 expression decreased in the exposure of fenofibrate in mantle cell lymphoma and prostate cancer cells as well [6,8]. However, in the present study, we detected notable decrease of Bcl-xl and increase of Bad but no significant changes in Bcl-2. Bad had been shown to bind more strongly to Bcl-xl than Bcl-2, and it could reverse the anti-apoptosis activity of Bcl-xl, but not that of Bcl-2 [29]. The phosphorylation of Bad by growth-factor-mediated signaling contributed to the cytoprotective function of Bcl-xl but not Bcl-2 [30]. These data showed a more intimate relationship between Bcl-xl and Bad than that between Bcl-2 and Bad, providing a strong support to our experimental results. Collectively, fenofibrate disrupts the net balance between pro- and anti-apoptosis in TNBC and then triggers caspase activation, leading to cell apoptosis ultimately.

Besides apoptosis, cell cycle arrest induced by fenofibrate in TNBC contributed to the anti-proliferation effect. Interestingly, the expression of p21 increased when the cells were exposed to fenofibrate for 6 hours, however, the effect disappeared when the exposure time lasted for 12 hours, implicating that the p21-mediated G0/G1 phase arrest might be an early event. Such G0/G1 phase arrest was accordance with the reports in prostate cancer [6], mantle cell lymphoma [8], endometrial cancer [17] and hepatocellular carcinoma [10]. The gene expression profile data in our research further confirmed the apoptosis and cell cycle arrest effects induced by fenofibrate.

The anti-proliferation and apoptosis-inducing effects of fenofibrate in TNBC were independent on PPAR-α status, which was also reported in B-cell lymphoma [5], prostate cancer [31], hepatocellular carcinoma [10], mantle cell lymphoma [8] and endometrial cancer [17]. However, the PPAR-α dependent mechanisms were used to explain the anti-cancer effects of fenofibrate in glioma [12], glioblastoma [7] and melanoma [14]. This paradoxical phenomenon might be due to the differences in tumor types or experimental conditions.

The further investigation shed light on the possible mechanisms of apoptosis induced by fenofibrate, showing that activation of NF-κB pathway played an important role. In the presence of fenofibrate, PDTC inhibited the accumulation of p65 in the nucleus and reversed the apoptosis effect. It is well known that NF-κB has bidirectional modulatory effects on cell apoptosis [32,33]. Consistent with our findings, several studies showed that up-regulation of NF-κB was associated with cyanide-induced apoptosis [34], thymocyte apoptosis [35], both paclitaxel- and doxorubicin-induced apoptosis [36,37], and acted as anti-oncogene [38,39]. However, there were a few reports indicating that down-regulation of NF-κB signaling was observed in fenofibrate-related apoptosis in lung cancer [15] and mantle cell lymphoma [8]. Unlike our experiment, Liang et al. pretreated cancer cells with TNF-α, which artificially activates NF-κB signaling [15]. The work by Zak et al. only showed that fenofibrate could down-regulate the NF-κB signaling [8]. Combined together, fenofibrate kills cancer cells possibly via NF-κB signaling status.

Cyto-protective pathways, such as Akt1 and/or Erk1/2 pathways might also be involved in anti-tumor effects of fenofibrate in TNBC. Inhibition of Akt and/or Erk1/2 pathways led to apoptosis and cell cycle arrest [40-42]. It has been reported that fenofibrate induced attenuation of Akt and/or Erk1/2 activity in prostate cancer [6], hepatocellular carcinoma [10], melanoma [14], medulloblastoma [16] and glioma [12]. Inhibition of angiogenesis and migration by fenofibrate was related to the decreased Akt [43,44]. Akt activation might either inhibit apoptosis by phosphorylation of Bad [45], or lead to cell cycle arrest by down-regulation of p27/Kip1 [46] and p21 [47]. Inhibition of PI3K activity, leading to inhibition of Akt, induced G0/G1 phase cell cycle arrest accompanied by the decreased expressions of Cyclin D1 and Cdk4 [48]. It had been described that cross-talk existed between Akt and Erk cascades [49]. Thus, synergistic effects of Akt and Erk with fenofibrate treatment might be more potent than either pathway alone involved.

Last we asked whether the effective drug concentration found in our experiments was easy to achieve in cancer patients because the IC_50_ of fenofibrate for MDA-MB-231 cells seemed to be higher. Willson et al. found that the human half maximal effective concentration (EC_50_) of fenofibrate was about 30 μM, and all PPAR-α agonists demanded such high micromolar concentration to activate PPAR-α ligands, which might explain why high doses were clinically needed [50]. Therefore, the concentration of fenofibrate used in our study was within the range achieved in patients with hypertriglyceridaemia and mixed dyslipidaemia who were treated with common dose of fenofibrate. A safety issue was another concern. Fenofibrate had the least effects on human breast epithelial cells (Figure 1A and Additional file 1B) and the data from the xenograft mouse model provided the evidence that such doses of fenofibrate were safe and had little side effects on hematologic, hepatic and renal functions.

Unlike other new developing anti-TNBC drugs, fenofibrate had been approved by the Food and Drug Administration for clinical use in patients with hypertriglyceridaemia and mixed dyslipidaemia for decades. Besides, fenofibrate was renal protective in doxorubicin-induced glomerular injury [51] and cisplatin-induced proximal tubule cell death [52]. However, mechanisms of sensitivity differences among breast cancer molecular subtypes and synergetic effects of fenofibrate with chemotherapy drugs in TNBC remain unclear in our study, further research will be paramount to unravel the mysteries.

## Conclusions

In conclusion, our results showed that fenofibrate was able to induce apoptosis *in vitro* and *in vivo* in TNBC involving the activation of NF-κB pathway, which might widen the anti-cancer spectrum of fenofibrate. The safety, convenience and affordability of fenofibrate make it a promising cancer therapeutic agent in TNBC.

## Abbreviations

TNBC: Triple-negative breast cancer; PPAR-α: Peroxisome proliferator-activated receptor alpha; PDTC: Pyrrolidine dithiocarbamate; IC50: Half maximal inhibitory concentration; WBC: White blood cell; HGB: Hemoglobin; PLT: Platelet; AST: Aspartate transaminase; ALT: Alanine transaminase; BUN: Blood urea nitrogen.

## Competing interests

The authors declare that they have no competing interests.

## Authors’ contributions

HXC, ZQL, ZJ and YG made contributions to the conception, design and analysis of data. LT carried out most of the experiments and drafted the main manuscript. HXC, ZQL and ZJ revised the manuscript. SZM and LJM provided technical supports. FMH helped with TUNEL. NC and WZH discussed the data. All authors have read and approved the manuscript for publication.

## Pre-publication history

The pre-publication history for this paper can be accessed here:

http://www.biomedcentral.com/1471-2407/14/96/prepub

## Supplementary Material

Additional file 1 **The effect of fenofibrate on less sensitive breast cancer cells and human breast epithelial cells.(A)** Fenofibrate induced apoptosis of BT549 cells. **(B)** Fenofibrate barely induced apoptosis of MCF-10A cells, which were human breast epithelial cells. **(C)** Fenofibrate arrested cell cycle of MDA-MB-468 cells at G0/G1 phase. The experiments were repeated three times and the representative ones of those results were shown. Click here for file

Additional file 2 **The inhibition of PPAR-α by GW6471.** 5 μM GW6471 significantly inhibited PPAR-α activity, decreasing the ANGPTL4 expression of MDA-MB-231 cells, which was a PPAR-α classic target gene. Target mRNA level was normalized to GAPDH mRNA level. The result was expressed as fold change (±SEM) relative to the control. *P < 0.05, ***P < 0.001. Angiopoietin-like 4 = ANGPTL4, Feno = fenofibrate. For details of real-time PCR assay, see Methods of Additional files in Additional file 3. Click here for file

Additional file 3 **Methods of Additional files.** The method of quantitative real-time PCR to measure the ability of GW6471 inhibiting PPAR-α activity. Click here for file
