# Supplementary figures and images for: Fenofibrate induces apoptosis of triple-negative breast cancer cells via activation of NF-κB pathway
Source: BMC Cancer. 2014 Feb 16;14:96. doi: 10.1186/1471-2407-14-96 (PMC4015735; doi:10.1186/1471-2407-14-96)

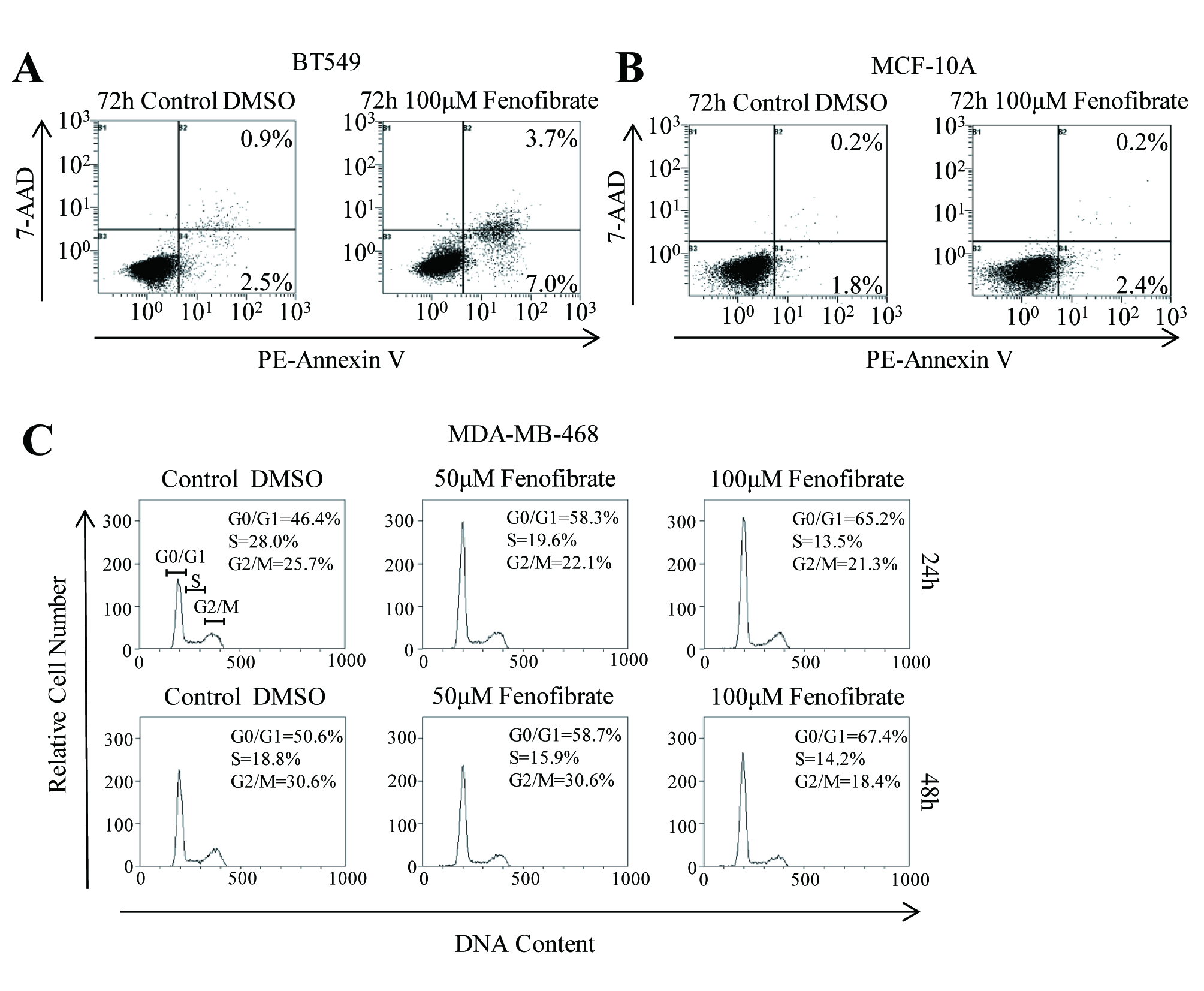

Supplement: Additional file 1 — The effect of fenofibrate on less sensitive breast cancer cells and human breast epithelial cells.(A) Fenofibrate induced apoptosis of BT549 cells. (B) Fenofibrate barely induced apoptosis of MCF-10A cells, which were human breast epithelial cells. (C) Fenofibrate arrested cell cycle of MDA-MB-468 cells at G0/G1 phase. The experiments were repeated three times and the representative ones of those results were shown. [file 1471-2407-14-96-S1.tiff]

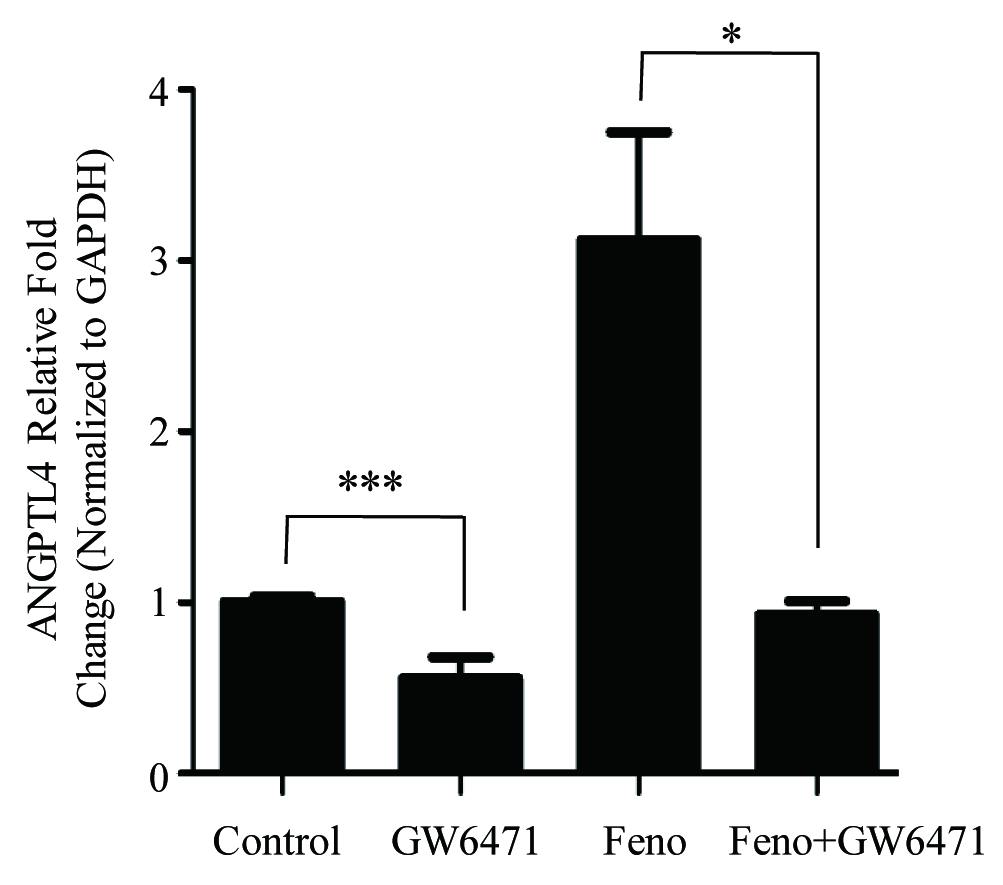

Supplement: Additional file 2 — The inhibition of PPAR-α by GW6471. 5 μM GW6471 significantly inhibited PPAR-α activity, decreasing the ANGPTL4 expression of MDA-MB-231 cells, which was a PPAR-α classic target gene. Target mRNA level was normalized to GAPDH mRNA level. The result was expressed as fold change (±SEM) relative to the control. *P < 0.05, ***P < 0.001. Angiopoietin-like 4 = ANGPTL4, Feno = fenofibrate. For details of real-time PCR assay, see Methods of Additional files in Additional file 3. [file 1471-2407-14-96-S2.tiff]
