# Supplementary material for: Fenofibrate induces apoptosis of triple-negative breast cancer cells via activation of NF-κB pathway
Source: BMC Cancer. 2014 Feb 16;14:96. doi: 10.1186/1471-2407-14-96 (PMC4015735; doi:10.1186/1471-2407-14-96)
Supplement: Additional file 3 — Methods of Additional files. The method of quantitative real-time PCR to measure the ability of GW6471 inhibiting PPAR-α activity. [file 1471-2407-14-96-S3.pdf]

Methods of Additional files

Quantitative real-time PCR

To measure the ability of GW6471 inhibiting PPAR-α activity, quantitative real-time PCR was performed to assess angiopoietin-like 4 (ANGPTL4) expression, which was the PPAR-α classic target gene [1, 2]. Total RNA was isolated by TRIzol (Invitrogen) after MDA-MB-231 cells treated with DMSO or 50 μM fenofibrate with or without 5 μM GW6471 for 24 h. First strand cDNA was synthesized from 1 μg of total RNA of each cell sample using PrimeScript™ RT reagent Kit (TaKaRa, Code No. RR047A). The cDNAs were used as templates for quantitative real-time PCR reactions. The PCR primers used were as follows: the forward primer, GGCTCAGTG GACTTCAACCG, the reverse primer, CCGTGATGCTATGCACCTTCT for ANGPTL4; the forward primer, ACAACTTTGGTATCGTGGAAGG, the reverse primer, GCCATCACGCCACAGTTTC for GAPDH. Sequences were 5' to 3'. Quantitative real-time PCR were carried out using SYBR® Green Real time PCR Master Mix (TOYOBO, Code No.QPK-201) according to manufacturer's instructions. The analysis was performed using mastercycler ep realplex^4 (Eppendorf). Amplification specificity was verified by visualizing PCR products on an ethidium bromide-stained 3% agarose gel. Gene expression was normalized to GAPDH and change in gene expression was measured relative to the control. All experiments were repeated in triplicate.

References:

1. Heinaniemi M, Uski JO, Degenhardt T, Carlberg C: **Meta-analysis of primary target genes of peroxisome proliferator-activated receptors**. *Genome Biol* 2007, **8**(7):R147.

2. Rakhshandehroo M, Knoch B, Muller M, Kersten S: **Peroxisome proliferator-activated receptor alpha target genes**. *PPAR Res* 2010, **2010**.
